# Magnetic Resonance Imaging (MRI) Evaluation and Classification of Vascular Malformations

**DOI:** 10.7759/cureus.67475

**Published:** 2024-08-22

**Authors:** Rajesh Kuber, Prajakta P KirdatPatil, Aryaman Dhande, Rahul Mane, Pushkar Kumar

**Affiliations:** 1 Radiodiagnosis, Dr. D. Y. Patil Medical College, Hospital and Research Centre, Dr. D. Y. Patil Vidyapeeth, Pune, IND; 2 Radiodiagnosis, Axis Diagnostic Centre, Sangola, IND

**Keywords:** imaging modalities, pediatric radiology, venous malformations, soft tissue lesions, magnetic resonance imaging (mri), vascular malformations

## Abstract

Introduction

Vascular malformations of the soft tissues are a diverse collection of lesions frequently encountered in clinical practice. Vascular malformations are rare and complex abnormalities that affect both children and young adults. Low-flow malformations are more common in children and often become symptomatic in later years. These malformations are common causes of soft tissue masses in children and can affect any part of the body at any age. Significant advancements in the management of these conditions have been made due to the implementation of a comprehensive binary categorization system, which classifies vascular abnormalities into tumors and malformations based on their clinicopathological characteristics. Imaging, particularly magnetic resonance imaging (MRI), plays a crucial role in the accurate identification, localization, and classification of these lesions, aiding in the development of appropriate treatment plans.

Materials and methods

This prospective study was conducted at Dr. D. Y. Patil Medical College, Hospital and Research Centre, Pimpri, Pune, from August 2022 to June 2024. Fifty patients of all age groups with clinically suspected soft tissue vascular malformations were included. MRI was performed using a MAGNETOM Vida (3T) Scanner (Siemens Healthcare Private Limited, Mumbai, India), and ultrasound was used as an adjunct. Institutional Ethics Committee clearance and informed consent were obtained. The study employed various MRI sequences, including T1-weighted imaging (T1WI) fast spin echo (FSE), T2-weighted imaging (T2WI) FSE, short tau inversion recovery (STIR), T2-weighted gradient recalled echo (GRE), pre-contrast fat-saturated T1WI, 3D post-contrast T1WI, diffusion-weighted imaging (DWI), and ANGIO TWIST (time-resolved angiography with interleaved stochastic trajectories) ISO.

Results

The study included 50 patients, with a male predominance of 28 (56%). The mean age was 22.13 years, and the average duration of vascular malformations was 32.94 months. The swelling was present in 43 (86%) of patients, and 35 (70%) had superficial lesions. MRI findings revealed hypointensity on T1 imaging in 40 (80%) patients and hyperintensity on T2 imaging in 49 (98%) cases. STIR sequences showed hyperintensity in all patients. The most common type of vascular malformation was slow-flow 46 (92%), with venous malformations being the most prevalent 39 (78%).

Conclusion

MRI is a valuable imaging modality for the evaluation and classification of vascular malformations, providing detailed information on lesion extent and involvement of surrounding tissues. The findings support the use of MRI as a primary tool in the assessment of vascular malformations, with ultrasound serving as a useful adjunct in certain cases. Further studies with larger sample sizes are recommended to validate these findings and refine imaging protocols.

## Introduction

Soft tissue vascular malformations, though rare, affect less than 1% of the population and are a common cause of soft tissue masses in children. While less prevalent than hemangiomas, which impact 2-3% of newborns, these anomalies can occur at any age and in various body locations [1]. Recent advancements in treatment are largely due to a refined classification system that distinguishes between tumors and malformations based on their clinicopathological features [1,2].

Accurately distinguishing between vascular malformations and tumors, such as hemangiomas, is crucial as their treatments differ significantly [3]. Most vascular anomalies can be identified through comprehensive clinical history and physical examination [4]. However, imaging is essential for confirming diagnoses, evaluating lesion size, and guiding treatment strategies. MRI and ultrasound are the main imaging techniques used. Ultrasound, being inexpensive and non-invasive, often does not require anesthesia, though its effectiveness may be limited for deep or complex lesions. MRI, on the other hand, provides superior soft tissue resolution without ionizing radiation, allowing a detailed assessment of lesions and their interaction with surrounding tissues [5,6]. MRI techniques, including flow-weighted and fat-saturated sequences [7], and the use of contrast enhancement are particularly useful for differentiating between lymphatic and venous malformations (VMs) [8,9]. Despite advances in imaging, there is a lack of literature on evaluating vascular malformations with MRI, especially in conjunction with ultrasound and CT. This study aims to address this gap by exploring how MRI, ultrasound, and CT can be used together to assess soft tissue vascular malformations. Findings could lead to more accurate diagnostics, improved treatment planning, and better patient outcomes while enhancing the understanding of these imaging modalities' roles and interactions in managing vascular anomalies.

The objective of this study was to analyze MRI characteristics of vascular malformations, classify lesions based on flow patterns, and utilize ultrasound and color Doppler to characterize tumors and assess vascularity as complementary tools to MRI where applicable.

## Materials and methods

Study design and setting

The Dr. D. Y. Patil Medical College and Hospital and Research Centre in Pimpri, Pune, Maharashtra, planned the current study as a prospective observational study that would run from August 2022 to June 2024. The study's goal was to assess and categorize vascular malformations using magnetic resonance imaging (MRI), a non-invasive imaging method renowned for its ability to image soft tissues with high resolution.

Study participants

We determined the sample size for this study using statistical methods to ensure adequate power for detecting meaningful differences and achieving reliable results. We calculated the required sample size based on an anticipated effect size, a confidence level of 95%, and a power of 80%. The study's sample size included a total of 50 participants based on the inclusion criteria. The study recruited participants with suspected soft tissue vascular malformations from all age groups. Thorough clinical examinations identified these cases, supplemented by X-ray and ultrasound results.

**Figure 1:**
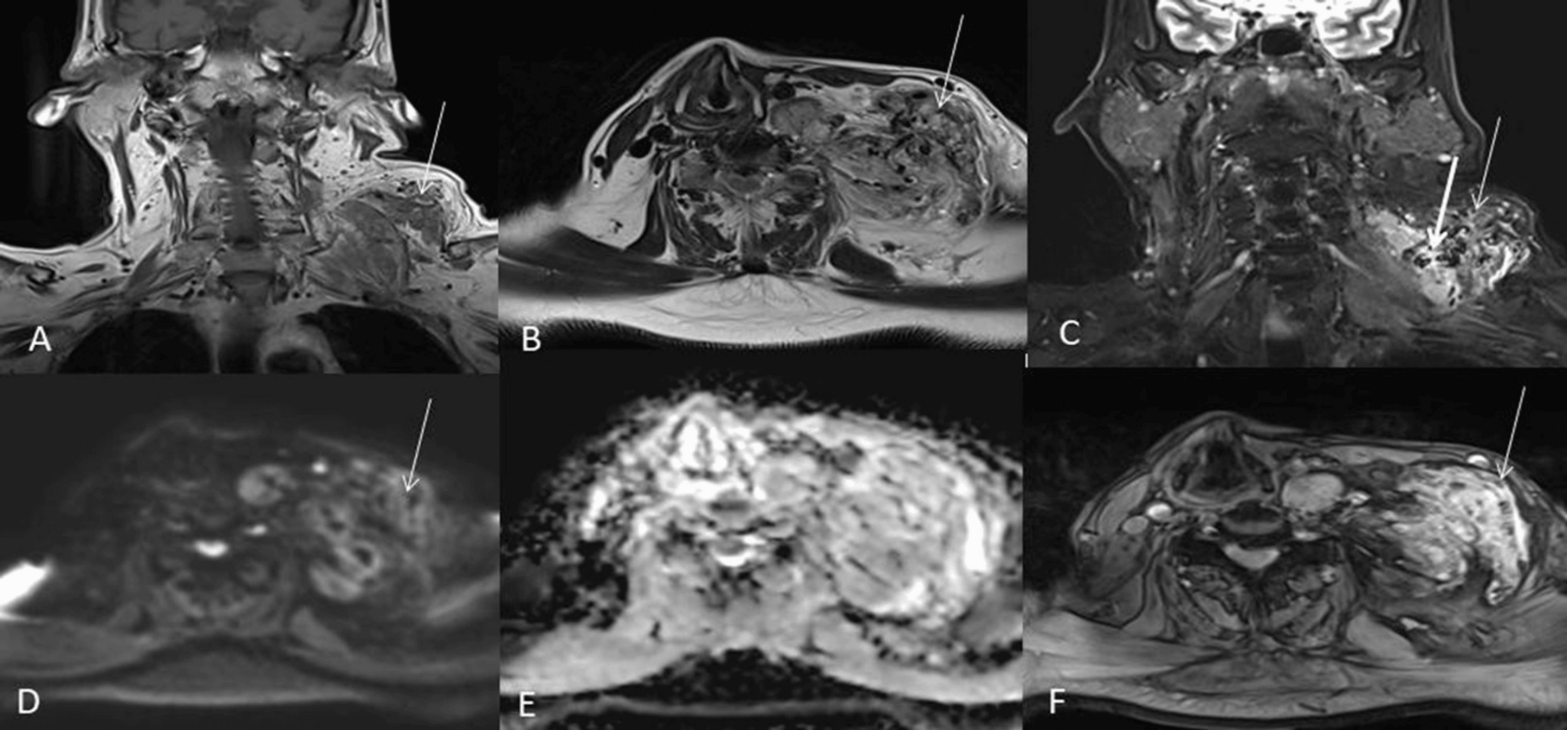
A male patient presented with left supraclavicular and neck swelling, predominantly in the deep subcutaneous and intermuscular plane. The swelling extended to the left paravertebral and posterior cervical space, causing a contour bulge in the left supraclavicular region. The lesion exhibited a predominantly isointense signal on T1-weighted imaging (T1WI) (A), with interspersed hyperintense areas likely representing fibro-fatty tissue. On T2-weighted imaging (T2WI) and short tau inversion recovery (STIR) sequences (B, C), the lesion appeared heterogeneously hyperintense. No diffusion restriction was observed (D, E). Multiple flow voids appearing hypointense were noted within the lesion on both T2WI and T1WI. Additionally, a few small low-signal intensity foci were seen within the lesion across all pulse sequences, showing blooming on gradient recalled echo (GRE), indicative of phleboliths (F).

Exclusion criteria were carefully defined to exclude patients with conditions that could interfere with MRI safety or imaging quality. These criteria included the presence of metallic hazards such as cardiac pacemakers or metallic foreign bodies, hemodynamic instability, claustrophobia, and the patient's uncooperative behavior. We obtained Institutional Ethics Committee (IEC) clearance before commencing the study and obtained informed written consent from all participants or their guardians, ensuring ethical compliance throughout the research process (Figure 1).

Variable measurement and data collection

We collected data using advanced MRI technology, specifically the MAGNETOM Vida MRI (3T) Scanner (Siemens Healthcare Private Limited, Mumbai, India) and Samsung HS70A with Prime (Samsung Electronics Private Limited, Delhi, India). We positioned the patients in a supine position, directing their heads toward the magnet (head first supine position), an optimal position for most MRI sequences. We employed specialized coils to enhance image resolution and diagnostic accuracy, depending on the anatomical location under evaluation. The MRI sequences employed in this study included T1-weighted imaging (T1WI) fast spin echo (FSE), T2-weighted imaging (T2WI) fast spin echo (FSE), short tau inversion recovery (STIR), T2-weighted gradient recalled echo (GRE), pre-contrast fat-saturated T1WI, 3D post-contrast T1WI, diffusion-weighted imaging (DWI), and ANGIO TWIST (time-resolved angiography with interleaved stochastic trajectories) ISO.

We meticulously selected and executed each sequence to capture specific aspects of vascular malformations, providing comprehensive anatomical and pathological details necessary for accurate classification and diagnosis.

Statistical analysis

We conducted data analysis using the Statistical Package for the Social Sciences (SPSS) (IBM SPSS Statistics for Windows, IBM Corp., Version 26.0, Armonk, NY). Quantitative data were analyzed to calculate descriptive statistics, such as mean (SD) and median (IQR), for continuous variables, while qualitative data were summarized using numbers and percentages. The findings were visually represented through appropriate statistical graphs, including pie charts and bar charts, to facilitate a clear and concise presentation of results.

Ethics statement: The Institutional Ethics Committee, Dr. D.Y. Patil Vidyapeeth, Pune, Maharashtra, approved DPU/R&R(M)/1188/161/2022.

## Results

Table 1 summarizes detailed patient characteristics observed in the study.

**Table 1:** Basic characteristics of study participants NF1, neurofibromatosis type 1

Characteristic	Frequency	Percentage
Male	28	56
Female	22	44
Age (years)	-	Value
Mean	-	22.13
Median	-	20.50
SD	-	15.95
IQR	-	11.75-32
Duration of vascular malformation (months)		
Mean	-	32.94
Median	-	18
SD	-	45.62
IQR	-	4-39
Swelling		
Present	43	86
Absent	7	14
Deep/superficial vascular malformations		
Superficial	35	70
Deep	15	30
Associated disorders		
None	44	88
Klippel-Trenaunay syndrome	2	4
NF1	3	6

Out of the 50 participants, there were 28 males (56%) and 22 females (44%), indicating a slight male predominance. The average age of the patients was 22.13 years, with a median age of 20.50 years, indicating that half were younger than this age. The standard deviation of 15.95 years reflected age variation within the group, while the IQR of 11.75 to 32 years indicated where the middle 25 (50%) of ages fell, showing variability around the median. Regarding vascular malformation, the average duration was 32.94 months, with a median of 18.00 months. The standard deviation of 45.62 months and an IQR of four to 39 months highlighted the variability in the duration of malformations observed among the patients. The swelling was present in 43 (86%) of patients, while 35 (70%) had superficial and 15 (30%) had deep vascular malformations. Most patients, 44 (88%), did not have associated disorders, with Klippel-Trenaunay syndrome observed in two (4%), neurofibromatosis in one (2%), and neurofibromatosis type 1 (NF1) in three (6%) cases.

Table 2 details the outcomes of MRI examinations conducted on the study participants.

**Table 2:** MRI findings among the study participants DWI, diffusion-weighted imaging; GRE, gradient recalled echo; NA, not applicable; STIR, short tau inversion recovery

Variables	Frequency	Percent
T1		
Hypointense	40	80
Hyperintense	4	8
Isointense	6	12
T2		
Hyperintense	49	98
Isointense	1	2
STIR		
Hyperintense	50	100
DWI		
Present	2	4
Absent	48	96
GRE		
Present	47	94
Absent	3	6
Contrast		
Present	28	56
Absent	6	12.0
NA	16	32.0

In T1WI, 80% of patients (40 out of 50) exhibited hypointensity, while 8% (four out of 50) showed hyperintensity, and 12% (six out of 50) had isointensity. T2WI revealed hyperintensity in 98% of cases (49 out of 50), with only 2% (one out of 50) displaying isointensity. STIR imaging detected hyperintensity in all patients (100%, 50 out of 50), indicating sensitivity to fluid accumulation and inflammation. DWI identified restricted diffusion in 4% of patients (two out of 50), suggesting cellular changes or ischemia, while 94% (47 out of 50) showed GRE features indicating hemorrhage or calcification; 56% of cases (28 out of 50) showed post-contrast enhancement, indicating active vascularization or inflammation, while 12% (six out of 50) did not exhibit enhancement due to contraindications. This table provided a comprehensive overview of the MRI findings, highlighting various tissue characteristics and anomalies detected among the study participants; 56% (28 out of 50 patients) showed improvement after receiving contrast. Contrasts typically show areas of active vascularization or inflammation within vascular malformations.

**Figure 2:**
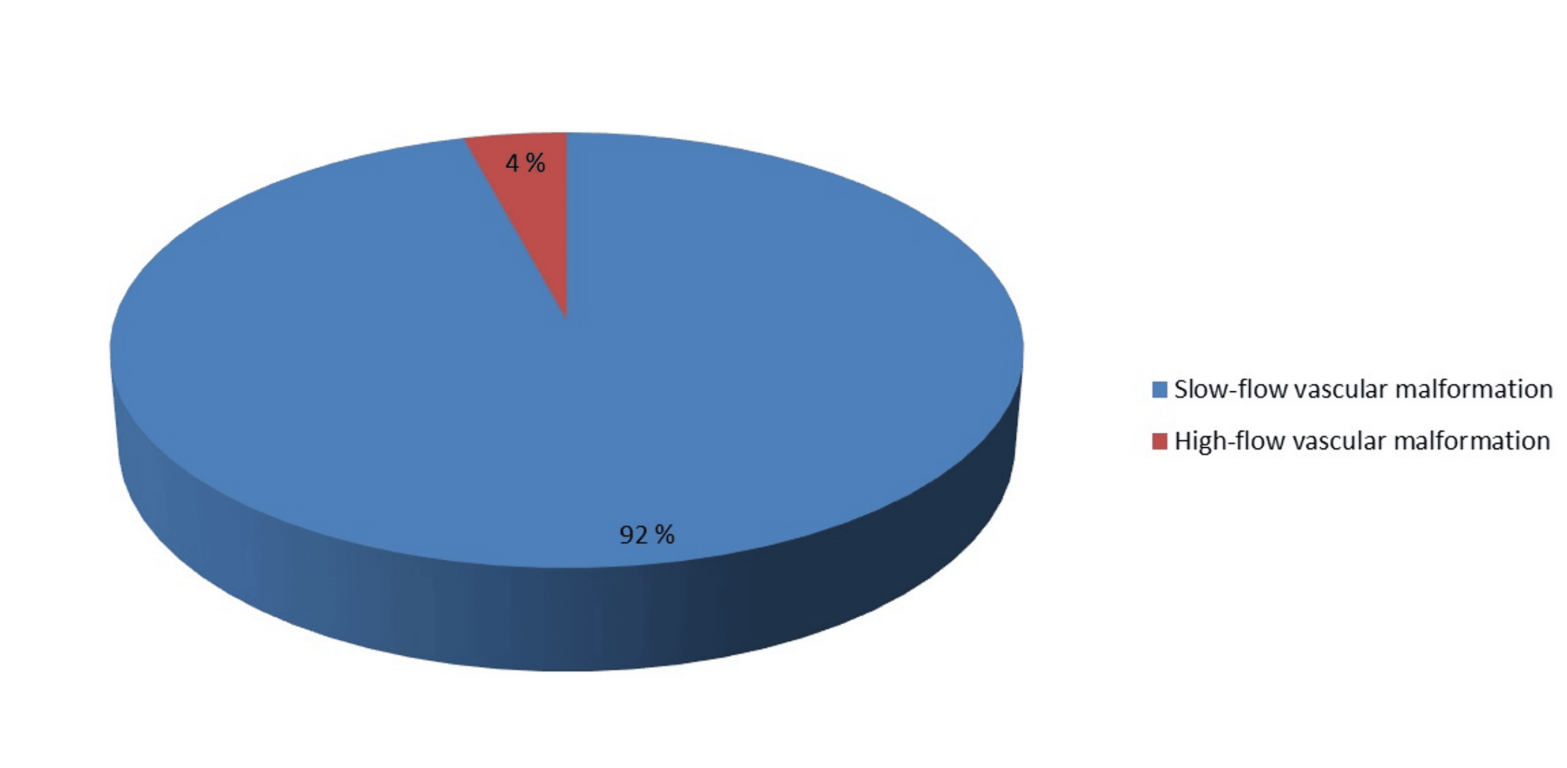
Pie chart showing the classification of vascular malformations.

The majority (92%, 46 out of 50 patients) were diagnosed with slow-flow vascular malformations, indicating predominant abnormalities characterized by sluggish blood flow. On the other hand, high-flow vascular malformations were less common, occurring in only 8% of patients (four out of 50). Abnormal arterial-venous connections, which cause high blood flow rates, mark these abnormalities. Of the specific types of vascular malformations observed, VMs were the most common, affecting 78% (39 out of 50 patients), followed by arterio-VMs (AVMs) in 8% (four out of 50 patients), lymphatic malformations in 6% (three out of 50 patients), and veno-lymphatic malformations in 6% (three out of 50 patients). Figure 2 illustrates the various types and locations of vascular anomalies identified in the study group. Clinical assessment and diagnostic imaging methods can detect a wide range of vascular malformations.

**Figure 3:**
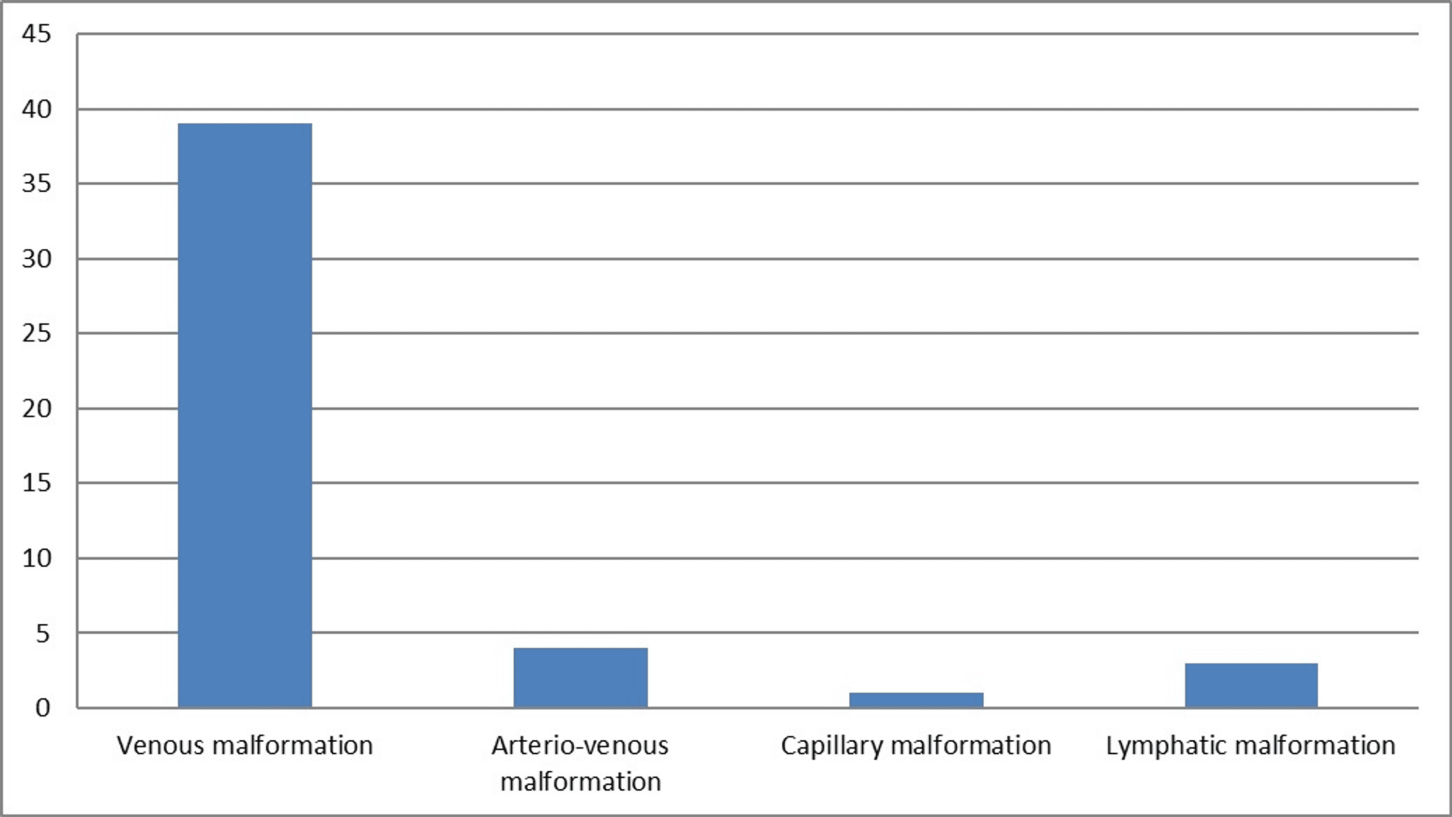
Bar diagram showing the distribution of vascular malformations.

Figure 3 summarizes the types and prevalence of vascular malformations observed in the study.

It shows that 46 (92%) of the patients had slow-flow vascular malformations, which means that blood flow was low, and four (8%) had high-flow malformations, which means that the connections between the arteries and veins were not working properly, which meant that blood flow was high. VMs were the most common type, occurring in 39 (78%) cases, followed by AVMs in four (8%) cases, capillary malformation in one (2%) case, lymphatic malformation in three (6%) cases, and veno-lymphatic malformation in three (6%) cases. This provides a concise overview of the distribution of vascular anomalies diagnosed through clinical and imaging assessments in the study cohort.

Table 3 presents two key aspects related to the study participants: USG correlation and comorbidities.

**Table 3:** USG correlation and comorbidities among the study participants

Characteristic	Frequency	Percent
Yes	7	14
No	43	86
Comorbidities		
None	45	90
Hypothyroidism	1	2
Lymphadenitis	1	2
Neurofibromas, axillary freckling, café au lait spots	1	2
Stroke	1	2
Varicose veins	1	2

Concerning USG correlation, 14% of the patients (n = 50) had a link between the USG findings and the MRI results. This shows that these two diagnostic methods are consistent when looking for vascular malformations. The majority, 86% (43 out of 50 patients), did not show such a correlation, suggesting potential differences or limitations between the two imaging techniques in specific cases. In terms of comorbidities, we identified 10% of the patients (five out of 50) as having additional health conditions alongside their vascular malformations. These included hypothyroidism, lymphadenitis, neurofibromas with axillary freckling and café au lait spots, stroke, and varicose veins, each accounting for one (2%) of the study cohort.

## Discussion

The study's goal was to look at the features, imaging results, and other health problems that are common in people with vascular malformations using MRI and see if these results were related to clinical evaluations and USG results. The International Society for the Study of Vascular Anomalies (ISSVA) classification serves as a globally recognized framework for categorizing all vascular malformations and tumors using consistent nomenclature [10,11]. The study cohort comprised 50 patients, with a slight male predominance of 28 (56%). The average age was 22.13 years, and the median age was 20.50 years, indicating that half of the patients were younger than 20.50 years. The broad age range, as evidenced by the standard deviation of 15.95 years and the IQR of 11.75 to 32 years, suggests that vascular malformations affect a diverse age group. The average duration of vascular malformations was approximately 33 months, with a median duration of 18 months, reflecting variability in disease onset and progression.

Swelling was a common clinical feature observed in 43 (86%) patients. The study revealed a higher prevalence of superficial vascular malformations in 35 patients (70%) compared to deep ones in 15 patients (30%), suggesting that detecting and reporting superficial malformations is easier. Most patients, 44 (88%), did not have associated disorders, indicating that vascular malformations often occur as isolated conditions. However, a small percentage had comorbid conditions such as Klippel-Trenaunay syndrome, neurofibromatosis, and NF1. Dhagat et al. looked at 52 cases of low-flow vascular malformations and found that 63% were ventricular malformations, 21% were lymphatic malformations, and the rest were veno-lymphatic malformations [12]. Some cases showed hypointensity on T1, while a similar proportion showed hyperintensity on T2. In our study, over 90% presented with low-flow vascular malformations, particularly higher AVMs compared to Dhagat et al., Breugem et al. found 85% of cases with low-flow lesions, similar to our majority (92%) [13]. In 27 patients, Rijswijk et al. documented 83% sensitivity and 95% specificity for peripheral vascular malformations using dynamic MRI [14].

Compared to our study, Hohn et al.'s study had a higher percentage of high-flow malformations (26.8%), probably because they included a larger age group [15]. They also reported 17%, which is in line with our study's 12% lymphatic involvement. Compared to our study, Habib et al. included 60 cases of vascular anomalies, with a mean participant age of 12.2 ± 9.4 years [16]. There were 74% females. Of the cases, 45% had VMs, 15% had lymphatic malformations, and 2% had Klippel-Trenaunay-Weber syndrome. While the diagnostic accuracy of both modalities was comparable, MRI showed better depth detection than ultrasound.

Numerous studies continue to reinforce current findings; on T1-weighted images, hypointensity was the most common finding, 40 (80%), while T2-weighted images predominantly showed hyperintensity, 49 (98%). The STIR sequence detected hyperintensity in all patients, indicating its high sensitivity to fluid accumulation and inflammation. In 47 (94% of the patients) cases, GRE sequences revealed features suggestive of hemorrhage or calcification, while 28 (56% of the cases) cases showed post-contrast enhancement, indicating active vascularization or inflammation [12,17-18]. We classified the majority of the malformations, 46 cases (92%), as slow-flow, characterized by sluggish blood flow. High-flow malformations, characterized by abnormal arteriovenous connections, were less frequent (4%). VMs were the most common type, occurring in 39 cases (78%), followed by AVMs in four cases (8%), lymphatic malformations in three cases (6%), and veno-lymphatic malformations in three cases (6%). This distribution highlights the predominance of VMs in the study cohort.

The study also examined the correlation between USG and MRI findings. Only seven (14% of the patients) showed a correlation between these imaging modalities, suggesting potential limitations of USG for certain types of vascular malformations, although it remains the first choice for initial evaluation [19]. Accurately describing the shape, structure, and function of cerebral vascular malformations through a range of imaging studies is key to figuring out the best way to treat them, and imaging evaluation of these lesions is still needed for new therapeutic efforts [20].

First, Doppler-type ultrasound uses morphological and functional data to identify entities such as VMs and AVMs, with low flow for VMs and high flow for AVMs [21]. However, it is ineffective for large, deep bone lesions [8] or deformities such as leptomeningeal angioma associated with Sturge-Weber syndrome (SWS), capillary cavernous telangiectasia (CCT), and capillary angioma (CA). We advise MRI as the primary imaging modality in these situations due to the inconclusive results of ultrasound [21]. MRI protocols commonly include T1 and T2 sequences, except in cases involving CAs, which prefer GRE T2 due to angiographically concealed lesions [22].

Comorbid conditions were present in five (10%) patients, including hypothyroidism, lymphadenitis, neurofibromas, stroke, and varicose veins, highlighting the need for a comprehensive clinical evaluation in patients with vascular malformations.

## Conclusions

MRI is crucial for characterizing vascular malformations due to its detailed imaging capabilities. T2-weighted images reveal hyperintensity, which helps determine the extent of the malformation and its impact on surrounding tissues. Similarly, hyperintensity observed in STIR sequences indicates high fluid content and inflammation, which is common in these conditions. GRE sequences further enhance feature visibility, aiding in comprehensive evaluation and clinical decision judgment. Post-contrast enhancement provides additional detail, highlighting the complexity of these malformations and underscoring the importance of precise imaging. MRI's advanced capabilities offer essential insights into the imaging and clinical features of vascular malformations, reinforcing the need for ongoing research and multidisciplinary care to advance the understanding and treatment of these challenging conditions.
